# Assessing multidimensional student engagement in studio-based design learning: a traceable multimodal framework for teacher-led inquiry

**DOI:** 10.3389/fpsyg.2026.1903940

**Published:** 2026-08-17

**Authors:** Yunyi Meng, Zhentao Wang, Bo Ruan

**Affiliations:** 1 School of Art, Ningbo City College of Vocational Technology, Ningbo, Zhejiang, China; 2 Lingyang Intelligent Technology Co., Ltd., Hangzhou, Zhejiang, China

**Keywords:** educational psychology, evidence-based teaching inquiry, explainable assessment, multimodal learning evidence, student engagement, studio-based design learning, teacher agency

## Abstract

**Introduction:**

Student engagement is a central construct in educational psychology, but it is difficult to assess in studio-based design learning because participation, emotion, cognitive effort, self-regulation, and design-process activity are distributed across classroom interaction, learning records, written reflection, and design artifacts.

**Methods:**

This study presents PD-EviMEA as a proof-of-concept and modular feasibility framework that organizes such traces into teacher-reviewable evidence for engagement assessment. It represents visual, behavioral, textual, and design-process information as learning evidence, estimates modality reliability and availability, and links each diagnostic statement to explicit evidence units rather than treating model outputs as self-sufficient judgments.

**Results:**

Across several public datasets, the results provide preliminary modular evidence that multimodal traces can support engagement recognition, that learning logs and design-process sequences can function as contextualized indicators of behavioral and cognitive engagement, and that evidence-constrained feedback can preserve alignment with human scoring criteria while adding traceability.

**Discussion:**

Because the datasets were not collected from a single product design studio and several analyses rely on proxy tasks, the findings should be interpreted as public-dataset-based feasibility evidence rather than validation of a deployable classroom assessment system. The study clarifies how multidimensional engagement may be operationalized in studio-based design contexts while preserving teacher agency, construct caution, and transparency in technology-supported assessment.

## 1 Introduction

Studio-based design learning is typically project-oriented, artifact-mediated, and socially interactive. In such learning environments, students are expected not only to attend class and complete assignments, but also to identify user needs, generate sketches, revise alternatives, construct computer-aided design models, explain design rationales, and respond to teacher or peer feedback. These activities make student engagement a central psychological and pedagogical construct for understanding how learners participate in, persist in, regulate, and take agency in design learning. The present study uses vocational product design as the motivating context, while addressing a broader problem in studio-based design education: how multidimensional engagement can be assessed when its observable evidence is distributed across classroom behavior, affective expression, cognitive effort, reflection, and artifact-making processes.

Educational psychology has commonly conceptualized student engagement as a multidimensional construct involving behavioral, emotional, and cognitive engagement (Fredricks et al., 2004; Bond et al., 2020). Related work on achievement emotions, self-regulated learning, and agentic engagement further indicates that engagement cannot be inferred from visible participation alone (Pekrun, 2006; Zimmerman, 2002; Reeve and Tseng, 2011). This point is especially important in studio learning. A student may appear quiet during a critique session while remaining cognitively engaged in design reasoning; another student may interact frequently with a digital platform while showing little evidence of sustained revision or reflective explanation. Therefore, the methodological challenge is not simply to collect more data, but to connect heterogeneous learning traces to educationally meaningful interpretations without treating computational predictions as psychological facts.

Recent studies in learning analytics, educational data mining, and multimodal learning analytics have made substantial progress in modeling learning participation, academic risk, and engagement-related states (Long and Siemens, 2011; Ferguson, 2012; Baker and Yacef, 2009; Gašević et al., 2015; Verbert et al., 2013; Blikstein, 2013; Ochoa, 2017; Mu et al., 2020). Public datasets have also supported research on online engagement recognition, classroom group engagement, learning behavior logs, rubric-aligned scoring, and design-process modeling (Kuzilek et al., 2017; Wu et al., 2024; Lu et al., 2025; The Learning Agency Lab, 2024; Lai et al., 2026; Willis et al., 2021). However, many existing approaches still face three limitations when considered from an educational psychology perspective. First, they often over-rely on behavioral or platform traces, which may under-represent emotional, cognitive, self-regulated, and agentic aspects of engagement. Second, they provide limited accounts of how design-process traces such as sketch revision, modeling continuity, and design-rationale refinement should be interpreted as contextualized evidence of engagement. Third, model outputs are often difficult for teachers to inspect, challenge, and translate into evidence-based teaching inquiry.

These limitations motivate the central task of this study: to develop a construct-informed and teacher-reviewable multimodal framework for assessing student engagement in studio-based design learning. The framework proposed in this paper, PD-EviMEA, organizes visual, behavioral, textual, and design-process traces into evidence units and evidence chains that can support engagement diagnosis and teaching inquiry. It does not treat design-process activity as a new universal psychological dimension. Rather, design-process evidence is interpreted as a domain-sensitive indicator that may express behavioral persistence, cognitive elaboration, self-regulation, and agentic participation in studio contexts. Methodologically, the framework first encodes available modalities, then applies reliability-gated fusion to reduce the influence of noisy or missing evidence, and finally constrains feedback generation so that diagnostic statements are linked to observable evidence units. Because no single public dataset contains complete vocational product design classroom data with multimodal traces, teacher ratings, engagement labels, and longitudinal outcomes, this study adopts a modular public-dataset-based feasibility strategy. The analyses examine whether different functional components of the framework can be supported by public evidence, rather than claiming direct classroom intervention validation.

Accordingly, this manuscript uses the terms proof-of-concept, modular feasibility evidence, and public-dataset-based analysis deliberately. The study does not claim that PD-EviMEA has already been deployed as a classroom assessment system, that the six datasets form one integrated learner cohort, or that public benchmark performance demonstrates educational effectiveness in a vocational product design studio. Instead, the purpose is to examine whether the core components needed for such a future system–multimodal engagement recognition, behavior-log evidence, design-process evidence, human-score alignment, evidence traceability, and ecological classroom recognition–can be supported under reproducible public-data conditions.

The main contributions of this study are threefold:

1. **A construct-informed account of engagement evidence in studio learning**. We connect multidimensional student engagement theory with domain-specific evidence in studio-based design learning, treating design-process traces as contextualized indicators of engagement rather than as a replacement for psychological interpretation.
2. **A teacher-reviewable multimodal assessment framework**. We propose reliability-gated multimodal fusion and evidence-chain-constrained feedback so that noisy or missing modalities are handled cautiously and each diagnostic statement can be inspected against observable evidence units.
3. **A transparent proof-of-concept modular feasibility analysis**. We examine PD-EviMEA across multiple publicly available datasets without merging them at the raw-sample level. The experiments provide preliminary evidence for functional components of the framework, including multimodal engagement recognition, behavior-log proxy modeling, design-process evidence modeling, rubric-aligned scoring, expert-scoring proxy analysis, and ecological classroom engagement recognition.

**Figure 1:**
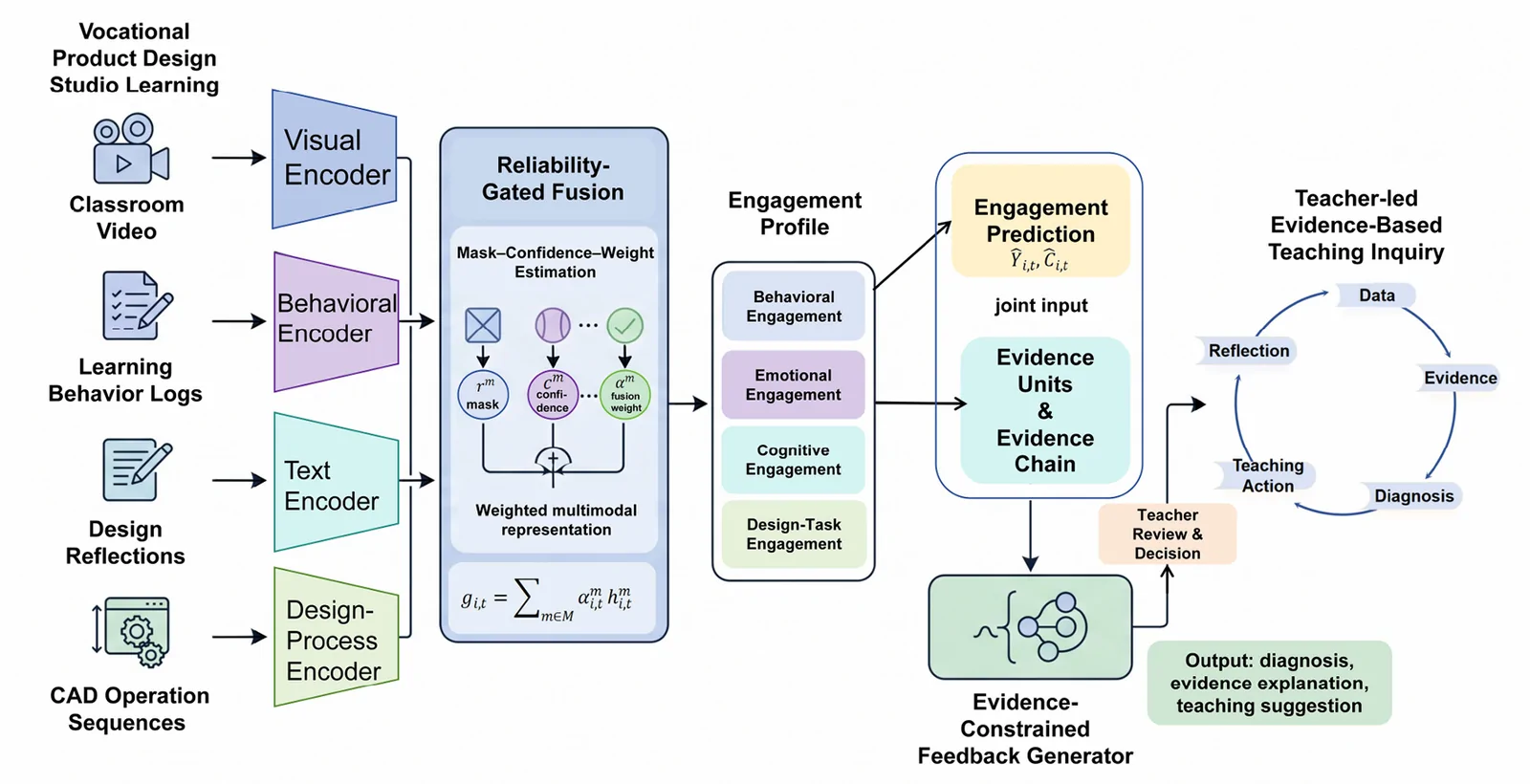
Overall workflow of PD-EviMEA for vocational product design studio learning. Classroom video, learning behavior logs, design reflections, and CAD operation sequences are first processed by modality-specific encoders and then integrated through reliability-gated fusion. The fused representation supports engagement profiling, engagement prediction, and evidence-unit construction. Evidence chains are passed to an evidence-constrained feedback generator and reviewed by teachers before informing evidence-based teaching inquiry through a data–evidence–diagnosis–teaching action–reflection cycle.

**Figure 2:**
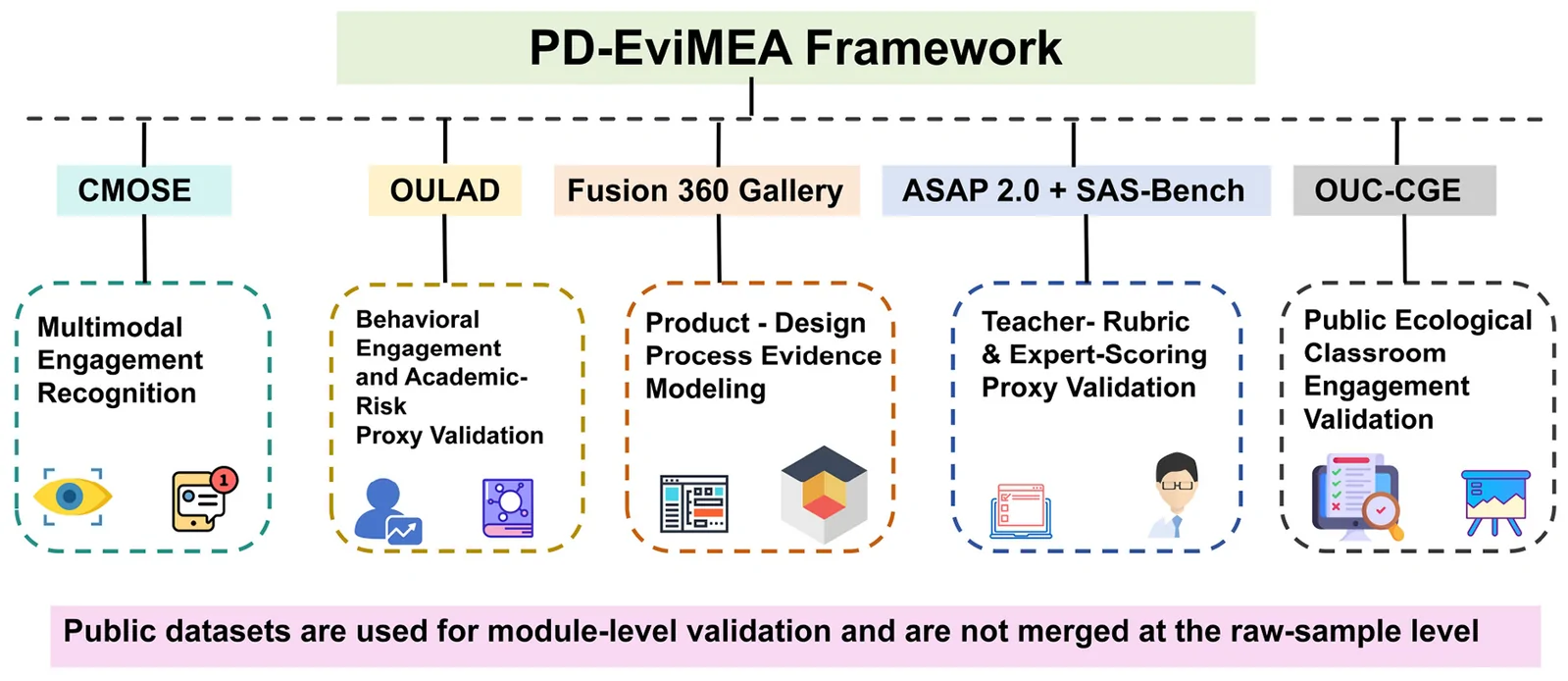
Modular public-dataset feasibility strategy for PD-EviMEA. CMOSE, OULAD, Fusion 360 Gallery, ASAP 2.0, SAS-Bench, and OUC-CGE are used to examine different framework components, including multimodal engagement recognition, behavioral-engagement and academic-risk proxy analysis, product-design process evidence modeling, teacher-rubric and expert-scoring proxy validation, and public ecological classroom engagement validation. The datasets are not merged at the raw-sample level, and the figure represents component-level feasibility analysis rather than full-system classroom validation.

Figure 1 summarizes the overall PD-EviMEA workflow, in which multimodal traces are encoded, fused through reliability-gated multimodal fusion, transformed into engagement profiles and evidence chains, and reviewed by teachers for evidence-based teaching inquiry. Figure 2 further illustrates the modular public-dataset feasibility strategy used in the experiments.

The remainder of this paper is organized as follows. Section 2 reviews prior work on student engagement assessment, multimodal learning analytics, evidence-based teaching inquiry, explainable educational AI, and design-process analytics. Section 3 presents the proposed PD-EviMEA framework, including evidence-unit construction, reliability-gated multimodal fusion, engagement prediction, and evidence-chain-constrained feedback generation. Section 4 reports the public-dataset analyses, datasets, evaluation metrics, benchmark comparisons, ablation studies, generalization analysis, explanation analysis, parameter analysis, and limitations. Section 5 discusses the implications of the proposed framework for studio-based design education and human-in-the-loop evidence-based teaching inquiry. Finally, Section 6 concludes the paper and outlines future research directions.

## 2 Theoretical and methodological background

### 2.1 Student engagement assessment and evidence-based teaching inquiry

Research on student engagement and evidence-based teaching inquiry can be broadly organized into four streams. The first stream concerns the theoretical conceptualization of student engagement. Early work treated engagement as students' participation in school activities and identification with school, while later studies established the widely used multidimensional view of behavioral, emotional, and cognitive engagement (Finn, 1989; Fredricks et al., 2004). Related studies on achievement emotions, self-regulated learning, and agentic engagement further show that engagement is not merely a behavioral state but also involves affective, cognitive, motivational, and self-directed processes (Pekrun, 2006; Zimmerman, 2002; Reeve and Tseng, 2011). This framework has been influential in educational psychology because it links observable participation, affective attachment, and deep learning strategies to academic development. Recent reviews further emphasize that student engagement remains a complex and context-sensitive construct, and that learning analytics studies often over-rely on behavioral traces while under-representing emotional and cognitive dimensions (Bond et al., 2020). The second stream is learning analytics and educational data mining. Datasets such as OULAD have enabled researchers to model students' virtual learning environment interactions, assessment behavior, and academic risk at scale (Kuzilek et al., 2017). These studies show that log data can provide useful indicators of learning participation, but they also reveal a conceptual limitation: clickstream behavior is not equivalent to engagement itself. The third stream is multimodal learning analytics, which extends learning analytics beyond logs by incorporating video, speech, text, posture, gaze, physiological signals, and collaboration traces (Blikstein, 2013; Ochoa, 2017; Mu et al., 2020). Recent benchmarks such as CMOSE and OUC-CGE have advanced this line of work by providing multimodal online engagement labels and authentic classroom group engagement videos, respectively (Wu et al., 2024; Lu et al., 2025). Recent multimodal learning analytics studies have also emphasized feedback, reflection, fairness, accountability, transparency, and ethics as design requirements rather than optional add-ons (Yan et al., 2024a; Jin et al., 2024; Caskurlu et al., 2025). The fourth stream concerns evidence-based teaching inquiry and human-in-the-loop educational AI. Prior studies have argued that AI-supported learning analytics should not merely automate prediction, but should support teachers' interpretation, pedagogical judgment, and iterative inquiry (Schildkamp, 2019; Khosravi et al., 2022). Recent discussions on generative AI in education further highlight the need to preserve teacher agency, transparency, accountability, and professional responsibility when algorithmic systems are used in assessment and feedback (Kasneci et al., 2023; Yan et al., 2024b; Miao and Holmes, 2023; Miao and Cukurova, 2024).

The present study differs from these prior works in three respects. First, it focuses on studio-based design learning, using vocational product design as a concrete context in which engagement is expressed not only through attendance, emotion, and platform behavior, but also through sketch iteration, modeling continuity, design revision, and reflective explanation. Second, rather than treating multimodal engagement recognition as an isolated prediction task, this study links engagement diagnosis to evidence-based teaching inquiry through a traceable evidence chain. Third, the proposed framework keeps teachers in the decision loop: the model produces evidence-grounded diagnoses and suggested teaching actions, whereas final pedagogical interpretation remains with the teacher.

### 2.2 Evidence-chain-constrained multimodal assessment and design-process evidence

Research directly related to the methodological innovation of this paper can be divided into four categories. The first category is multimodal fusion for engagement recognition. Traditional approaches usually adopt early fusion, late fusion, score-level averaging, attention-based fusion, or stacking strategies to combine visual, textual, acoustic, and behavioral representations (Baltrušaitis et al., 2019; Mu et al., 2020). Recent engagement recognition studies have improved model performance by using stronger video-text encoders, ordinal learning, and imbalance-aware training; for example, CMOSE introduced multimodal features and engagement labels to support online engagement detection (Wu et al., 2024). However, many fusion methods still assume that all modalities are available and equally reliable, which is often unrealistic in classroom settings where video occlusion, missing audio, sparse text, or incomplete behavior logs are common. The second category is explainable and trustworthy AI in education. Explainable learning analytics has emphasized the need to make predictive models interpretable to teachers and learners, yet explanations are often *post-hoc* importance scores rather than pedagogically usable evidence (Lundberg and Lee, 2017; Khosravi et al., 2022). Human-centered and teacher-complementary AI studies further stress that model outputs should be reviewable, contestable, and aligned with human decision-making workflows (Amershi et al., 2019; Holstein et al., 2019; Shneiderman, 2020). With the rapid development of large language models, recent studies have explored automated feedback, rubric-based scoring, and generative assessment support, but concerns remain regarding hallucination, weak traceability, data privacy, fairness, and insufficient alignment with human judgment (Kasneci et al., 2023; Yan et al., 2024b; Miao and Holmes, 2023; Miao and Cukurova, 2024). The third category is rubric-aligned scoring and expert-annotated assessment. ASAP 2.0 provides open-access essay scoring data for automated writing assessment, while SAS-Bench offers fine-grained short-answer scoring with step-wise scores and expert-annotated error categories (The Learning Agency Lab, 2024; Lai et al., 2026). These datasets are valuable for validating whether model feedback can remain aligned with human scoring criteria, but they do not directly address multimodal studio learning. The fourth category is design-process analytics. In design education, studio learning has long been understood as a reflective, iterative, and artifact-mediated process (Cross, 2006). The Fusion 360 Gallery dataset provides human CAD construction sequences and makes it possible to computationally model sketch-and-extrude operations, design-stage transitions, and construction complexity (Willis et al., 2021). Nevertheless, existing CAD sequence studies are usually concerned with geometric reconstruction or design automation, not with connecting design-process evidence to student engagement and teaching inquiry.

This study addresses these gaps by proposing an evidence-chain-constrained multimodal assessment framework for studio-based design learning. Unlike conventional fusion models, PD-EviMEA introduces reliability-gated fusion with modality masks and confidence estimates, so that missing or noisy modalities can be down-weighted rather than blindly concatenated. Unlike generic explainable AI methods, it represents observable traces as evidence units and constrains large-model feedback to cite these units before generating pedagogical diagnoses. Unlike previous engagement studies that mainly rely on generic classroom or online-learning indicators, this study incorporates design-process evidence as a domain-specific proxy for engagement-related persistence, cognitive elaboration, and agency, thereby aligning multimodal assessment with the learning characteristics of design education.

## 3 Materials and methods

### 3.1 Problem formulation

This study aims to support the assessment of student engagement in studio-based design learning by integrating heterogeneous learning traces into pedagogically interpretable evidence. Let $S=\left\{s_{1},s_{2},\ldots ,s_{N}\right\}$ denote a set of students or learning episodes, where *N* is the number of observed instances. A studio-learning process is divided into *T* temporal windows or learning episodes. For the *i*-th learner or group in the *t*-th episode, the multimodal observation is denoted as


$$\begin{array}{l} X_{i,t}=\left\{X_{i,t}^{v},X_{i,t}^{b},X_{i,t}^{l},X_{i,t}^{d}\right\}, \end{array}$$ (1)

where $X_{i,t}^{v}$ represents visual or audio-visual evidence, $X_{i,t}^{b}$ represents learning behavior logs, $X_{i,t}^{l}$ represents textual evidence such as reflections, explanations, or transcripts, and $X_{i,t}^{d}$ represents design-process evidence such as sketch iteration, CAD operations, design revisions, and modeling complexity. To preserve the distinction between the psychological construct and its observable indicators, the target engagement construct is defined separately from the design-process evidence layer. The engagement vector is


$$\begin{array}{l} Y_{i,t}^{E}=\left[y_{i,t}^{A},y_{i,t}^{B},y_{i,t}^{C}\right], \end{array}$$ (2)

where $y_{i,t}^{A}$, $y_{i,t}^{B}$, and $y_{i,t}^{C}$ denote affective, behavioral, and cognitive engagement, respectively. Contextualized design-process evidence is represented separately as


$$\begin{array}{l} P_{i,t}=\phi_{d}\left(X_{i,t}^{d}\right), \end{array}$$ (3)

where *P*_*i,t*_ summarizes observable features such as modeling continuity, iterative sketch modification, and design-rationale refinement. Thus, *P*_*i,t*_ may corroborate, qualify, or challenge an interpretation of the three engagement dimensions, but it is not a constitutive dimension of the student-engagement construct. This separation is retained in the prediction outputs, evidence units, and teacher-facing interpretation described below.

Table 1 summarizes how the framework connects educational psychology constructs with observable evidence. The table is intended to make the construct interpretation explicit and to prevent the multimodal features from being treated as direct substitutes for psychological engagement.

**Table 1:** Construct-informed mapping between engagement constructs and observable evidence in studio-based design learning.

Engagement construct	Educational-psychological meaning	Observable evidence used in the framework	Interpretive boundary
Affective engagement	Emotional involvement, interest, and affective connection with the learning activity	Facial or bodily participation cues, expressive classroom behavior, emotion-related language in reflection, or explanation	Observable affective cues do not fully reveal internal emotional experience
Behavioral engagement	Participation, effort, persistence, and task involvement	Attendance-related traces, learning behavior logs, classroom activity, task continuation, submission, or interaction records	Activity frequency is not equivalent to meaningful engagement
Cognitive engagement	Mental effort, strategy use, elaboration, reflection, and self-regulated learning	Reflective writing, design explanations, problem-solving traces, revision rationales, evidence of monitoring, and refinement	Cognitive engagement requires interpretation of task quality and learning context
Complementary design-process evidence layer	Domain-sensitive traces that may contextualize persistence, cognitive elaboration, self-regulation, or learner initiative	Sketch iteration, computer-aided design operation sequences, design-stage transitions, modeling continuity, and artifact modification	This evidence is complementary to, rather than constitutive of, the multidimensional engagement construct

To maintain terminological consistency, this article uses *engagement dimensions* to refer to educational-psychological constructs such as affective, behavioral, and cognitive engagement; *design-task evidence* to refer to observable design-process traces that may inform engagement interpretation in studio contexts; *evidence units* to refer to individual teacher-reviewable records linking a trace, time window, confidence value, and construct interpretation; and *evidence chains* to refer to ordered sets of evidence units used to support a diagnostic or teaching-inquiry statement. This distinction is important because the framework treats traces as evidence for interpretation, not as direct measures of internal psychological states.

For ordinal engagement recognition, the model predicts


$$\begin{array}{l} {ĉ}_{i,t}\in \left\{0,1,2,3\right\}, \end{array}$$ (4)

where the four levels correspond to very low, low, high, and very high engagement in the CMOSE setting. For ecological classroom feasibility analysis on OUC-CGE, the model predicts


$$\begin{array}{l} {ĉ}_{i,t}^{eco}\in \left\{0,1,2\right\}, \end{array}$$ (5)

The multimodal input, engagement targets, complementary design-process representation, and label spaces are formally defined in Equations 1–5 where the three levels correspond to low, medium, and high group engagement.

### 3.2 Overview of PD-EviMEA

The proposed framework, PD-EviMEA, consists of four components: modality-specific encoding, reliability-gated fusion, evidence-unit construction, and evidence-chain-constrained feedback generation. The overall logic follows a data–evidence–action process. First, heterogeneous learning traces are encoded into a common latent space. Second, the model estimates modality availability and reliability to reduce the influence of noisy or missing modalities. Third, observable traces are converted into evidence units, which are further organized into evidence chains. Finally, the evidence chains are used to support engagement diagnosis and teacher-facing teaching inquiry feedback. The overall framework is shown in Figure 1. This design keeps the teacher in the decision loop: the model provides traceable evidence and suggested pedagogical actions, while the teacher retains the final interpretive and instructional authority. At the current stage, this workflow specifies a proof-of-concept architecture and public-data evaluation strategy; it is not presented as a fully deployed classroom product.

Algorithm 1 summarizes the algorithmic procedure of PD-EviMEA. The pseudocode is included to make the data–evidence–action transformation explicit and to avoid presenting the framework as an unspecified combination of existing modules.

Algorithm 1 Pseudocode of PD-EviMEA. 
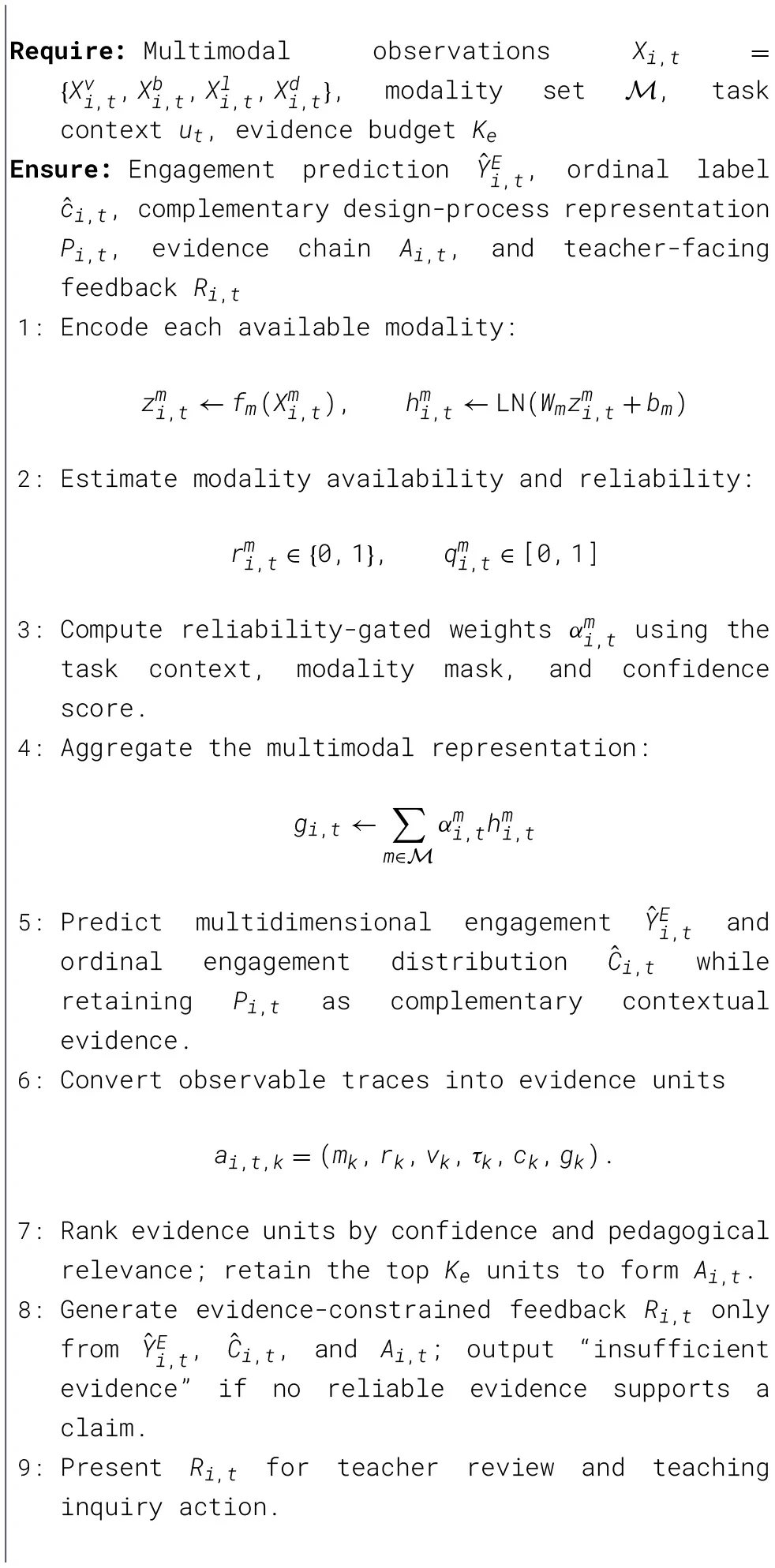

### 3.3 Modality-specific encoding

Each modality is encoded by a modality-specific encoder:


$$\begin{array}{l} z_{i,t}^{m}=f_{m}\left(X_{i,t}^{m}\right),\;m\in M, \end{array}$$ (6)

where M={v,b,l,d} denotes visual, behavioral, textual, and design-process modalities, respectively. The encoder *f*_*m*_(·) may be implemented using a pre-extracted feature encoder, a statistical feature extractor, a text vectorizer, or a sequence model, depending on the dataset and modality. For example, CMOSE uses I3D, BERT, and acoustic features; OULAD uses behavior-log statistics; Fusion 360 uses CAD operation sequence features; and OUC-CGE uses classroom-video visual statistics and metadata features.

To make heterogeneous modalities comparable, all encoded vectors are projected into a shared latent space:


$$\begin{array}{l} h_{i,t}^{m}=\text{LN}\left(W_{m}z_{i,t}^{m}+b_{m}\right), \end{array}$$ (7)

where *W*_*m*_ and *b*_*m*_ are trainable projection parameters, LN(·) denotes layer normalization, and $h_{i,t}^{m}\in {\mathbb{R}}^{d}$. In the implementation, *d* = 256 is used as the unified hidden dimension. When a modality is absent, the corresponding latent representation is not forced into the fusion module as a valid signal; instead, a modality mask is used to indicate whether the modality is available.

In interpretive terms, this stage does not yet diagnose engagement. It places heterogeneous traces in a comparable representation so that later stages can evaluate them jointly without assuming that a video cue, platform event, written reflection, and CAD operation have the same educational meaning.

### 3.4 Design-process evidence for product design learning

Product design studio learning differs from conventional lecture-based learning because engagement-related effort, persistence, and cognitive elaboration are often reflected in the process of artifact construction. Therefore, this study introduces design-process evidence as a domain-specific source for engagement interpretation. For a CAD sequence, the design-process input is represented as


$$\begin{array}{l} X_{i,t}^{d}\in {\mathbb{R}}^{L_{c}\times P_{c}}, \end{array}$$ (8)

where *L*_*c*_ is the length of the CAD operation sequence and *P*_*c*_ is the feature dimension of each operation. The operation-level features include operation type, sketch-and-extrude action, modification frequency, deletion frequency, temporal continuity, sequence length, transition pattern, and complexity-related statistics. The design-process encoder produces


$$\begin{array}{l} h_{i,t}^{d}=\text{LN}\left(W_{d}f_{d}\left(X_{i,t}^{d}\right)+b_{d}\right), \end{array}$$ (9)

The modality-specific and design-process encodings are specified in Equations 6–9 which serves as a domain-specific representation of design-task evidence. In this study, Fusion 360 Gallery Reconstruction is used to examine the feasibility of extracting such design-process evidence. It is important to note that CAD process features are treated as proxy evidence for engagement-related design activity rather than direct psychological labels. They form a complementary evidence layer: for example, repeated modification may support an interpretation of persistence or elaboration only when the task stage, artifact quality, and teacher knowledge make that interpretation plausible. The model therefore does not redefine engagement by adding design activity as another psychological dimension.

### 3.5 Reliability-gated multimodal fusion

Real classroom data often contain incomplete or noisy modalities. To address this issue, PD-EviMEA introduces a reliability-gated fusion mechanism. For each modality *m*, a binary availability indicator is defined as


$$\begin{array}{l} r_{i,t}^{m}\in \left\{0,1\right\}, \end{array}$$ (10)

where $r_{i,t}^{m}=1$ indicates that modality *m* is available and $r_{i,t}^{m}=0$ indicates that the modality is missing. A modality confidence score is further defined as


$$\begin{array}{l} q_{i,t}^{m}\in \left[0,1\right], \end{array}$$ (11)

where a higher value indicates a more reliable modality. For instance, a classroom video with severe occlusion receives a lower visual confidence score, while a complete behavior-log sequence receives a higher behavioral confidence score.

Given a task-context vector $u_{t}\in {\mathbb{R}}^{d}$, the reliability-gated modality weight is computed as


$$\begin{array}{l} \alpha_{i,t}^{m}=\frac{r_{i,t}^{m}\exp\left(\left(u_{t}^{⊤}h_{i,t}^{m}\right)/\sqrt{d}+\lambda q_{i,t}^{m}\right)}{\sum_{n\in M}r_{i,t}^{n}\exp\left(\left(u_{t}^{⊤}h_{i,t}^{n}\right)/\sqrt{d}+\lambda q_{i,t}^{n}\right)}, \end{array}$$ (12)

where λ controls the effect of modality confidence on fusion. The fused engagement representation is then calculated as


$$\begin{array}{l} g_{i,t}=\underset{m\in M}{\sum }\alpha_{i,t}^{m}h_{i,t}^{m},\;g_{i,t}\in {\mathbb{R}}^{d}. \end{array}$$ (13)

The availability, confidence, weighting, and fused-representation components of the reliability-gated mechanism are defined in Equations 10–13. Compared with direct concatenation, this mechanism avoids treating unavailable modalities as meaningful evidence. Compared with simple averaging, it allows the model to assign larger weights to modalities that are both informative and reliable.

For readers without a computational background, the practical purpose of this mechanism is to prevent a weak or missing source from dominating an engagement interpretation. The weights express the relative evidential contribution of available modalities in a given episode; they do not indicate the psychological importance of one engagement dimension over another.

### 3.6 Engagement prediction and training objective

The fused representation *g*_*i,t*_ is used for both multidimensional engagement estimation and ordinal engagement classification. The three-dimensional engagement vector is predicted by


$$\begin{array}{l} {Ŷ}_{i,t}^{E}=\sigma \left(W_{e}g_{i,t}+b_{e}\right), \end{array}$$ (14)

where ${Ŷ}_{i,t}^{E}\in {\left[0,1\right]}^{3}$ and σ(·) denotes the sigmoid function. The design-process representation *P*_*i,t*_ may contribute contextual evidence through the fused representation and subsequent evidence chain, but it is not reported as a fourth engagement score. The ordinal engagement distribution is predicted by


$$\begin{array}{l} {Ĉ}_{i,t}=\text{Softmax}\left(W_{c}g_{i,t}+b_{c}\right), \end{array}$$ (15)

where Ĉ_*i,t*_ is the predicted probability distribution over engagement levels.

The engagement prediction outputs and training objective are specified in Equations 14–16.


$$\begin{array}{l} L=L_{ord}+\beta L_{multi}+\gamma L_{align}+\delta L_{cal}+\eta L_{reg}, \end{array}$$ (16)

where $L_{ord}$ denotes ordinal classification loss, $L_{multi}$ denotes multidimensional engagement loss, $L_{align}$ denotes cross-modal alignment loss, $L_{cal}$ denotes calibration loss, and $L_{reg}$ denotes regularization. The coefficients β, γ, δ, and η control the relative importance of different objectives. In the public-dataset-based feasibility analyses, the exact loss components are adapted to the available labels of each dataset. For example, CMOSE supports multimodal engagement classification, OULAD supports behavioral-risk proxy prediction, Fusion 360 supports design-process sequence modeling, ASAP 2.0 supports rubric-aligned text scoring, SAS-Bench supports expert-scoring proxy analysis, and OUC-CGE supports ecological classroom engagement recognition.

In educational terms, the prediction layer provides a provisional pattern to be examined rather than a stand-alone judgment about a learner. The evidence-chain and teacher-review stages are therefore necessary for deciding whether the pattern is meaningful in the local task and classroom context.

### 3.7 Evidence units and evidence chains

To make the model output pedagogically interpretable, PD-EviMEA represents observable traces as evidence units. An evidence unit is defined as


$$\begin{array}{l} a_{i,t,k}=\left(m_{k},r_{k},v_{k},\tau_{k},c_{k},e_{k}\right), \end{array}$$ (17)

where *m*_*k*_ denotes the source modality, *r*_*k*_ denotes the engagement dimension supported by the evidence, *v*_*k*_ denotes the evidence value or textual content, τ_*k*_ denotes the timestamp or learning stage, *c*_*k*_ ∈ [0, 1] denotes the confidence of the evidence, and *e*_*k*_ denotes a human-readable explanation. For example, a design-process evidence unit may state that a student completed multiple CAD revisions after receiving teacher feedback, while a behavioral evidence unit may indicate delayed submission or sustained platform inactivity.

**Figure 3:**
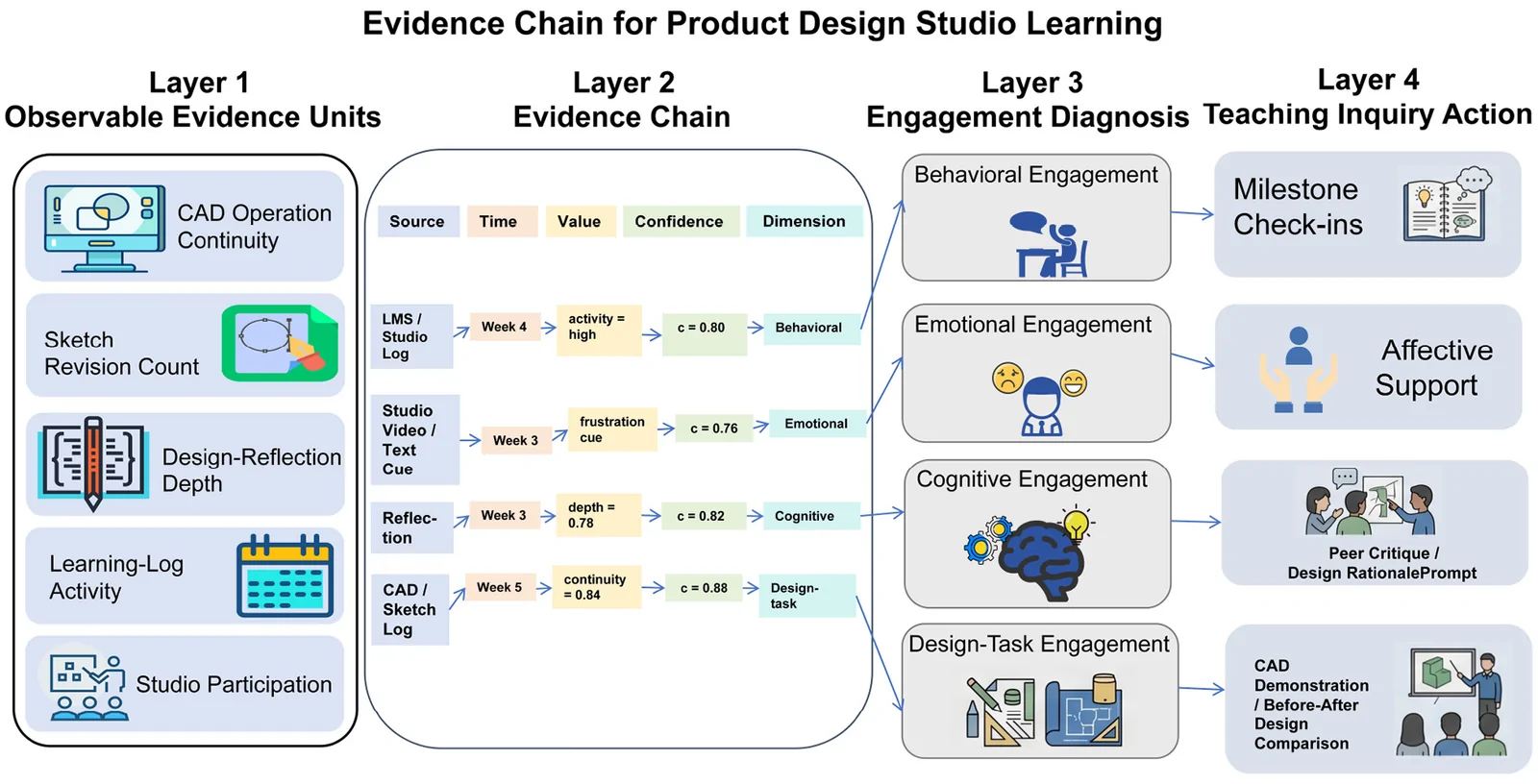
Evidence-chain structure for product design studio learning. Observable evidence units, such as CAD operation continuity, sketch revision count, design-reflection depth, learning-log activity, and studio participation, are organized into evidence chains with source, time, value, confidence, and engagement-dimension fields. These chains connect evidence units to engagement diagnosis and teacher-led inquiry actions, including milestone check-ins, affective support, peer critique or design-rationale prompts, and CAD demonstration or before–after design comparison. The diagram clarifies the teacher-reviewable record structure and workflow rather than introducing additional quantitative results.

The evidence chain for the *i*-th learner or group in episode *t* is defined as


$$\begin{array}{l} A_{i,t}=\left\{a_{i,t,1},a_{i,t,2},\ldots ,a_{i,t,K_{e}}\right\}, \end{array}$$ (18)

where *K*_*e*_ is the number of selected evidence units. The evidence units are ranked by confidence, relevance to the predicted engagement dimension, and diversity across modalities. In practice, only a limited number of high-confidence evidence units are presented to teachers to avoid cognitive overload. Figure 3 illustrates how observable evidence units are organized into evidence chains and translated into teaching inquiry actions.

In practical terms, an evidence chain functions like an auditable note: it shows the teacher what was observed, when it occurred, how confidently it was detected, and which interpretation it may support. Its purpose is to make the route from trace to pedagogical claim visible rather than to increase the apparent certainty of the prediction.

### 3.8 Evidence-chain-constrained feedback generation

The final output of PD-EviMEA is not only an engagement label, but also an evidence-grounded feedback item:


$$\begin{array}{l} R_{i,t}=\left(D_{i,t},E_{i,t},I_{i,t}\right), \end{array}$$ (19)

The evidence-unit, evidence-chain, and constrained feedback representations are defined in Equations 17–19 where *D*_*i,t*_ is the engagement diagnosis, *E*_*i,t*_ is the supporting evidence explanation, and *I*_*i,t*_ is the suggested teaching action. To reduce hallucination and unsupported pedagogical suggestions, the feedback generation process is constrained by the following rules:

1. each diagnostic statement must be supported by at least one evidence unit;
2. unsupported inferences must be marked as insufficiently evidenced;
3. suggestions must be aligned with the learning context of product design studio activities; and
4. model output is treated as decision support rather than final teacher judgment.

This constraint allows the system to produce feedback such as: “The available design-process evidence suggests sustained task work because the CAD sequence contains multiple revisions and stable operation continuity; however, the interpretation of cognitive engagement remains tentative because the design reflection provides limited explanation of user needs.” Such feedback can be directly discussed in evidence-based teaching inquiry meetings, where teachers inspect the evidence chain, revise the diagnosis if necessary, and decide on follow-up instructional actions.

Accordingly, the feedback generator is an evidence-organizing component rather than an autonomous adviser. Its educational value depends on whether the cited evidence helps a teacher formulate a proportionate response, request further information, or withhold judgment when the available traces are insufficient.

### 3.9 Human-in-the-loop teaching inquiry

The teacher-facing component aggregates individual evidence chains into class-level teaching inquiry evidence. For a class *j*, the engagement matrix is represented as


$$\begin{array}{l} E_{j}\in {\mathbb{R}}^{N_{j}\times T\times 3}, \end{array}$$ (20)

where *N*_*j*_ is the number of learners or groups in class *j*, *T* is the number of learning episodes, and the last dimension corresponds to affective, behavioral, and cognitive engagement. Complementary design-process records are maintained separately as *P*_*j*_ = {*P*_*i,t*_} and can be inspected alongside, but are not dimensions of, *E*_*j*_. Teaching strategies are encoded as


$$\begin{array}{l} S_{j,t}\in {\left\{0,1\right\}}^{Q}, \end{array}$$ (21)

where *Q* denotes the number of teaching strategy types, such as case introduction, demonstration, peer critique, individual guidance, design presentation, and reflective writing. The relation between teaching strategy and engagement change can be examined through


$$\begin{array}{l} E_{i,t+1}^{r}=\theta_{0}+\theta_{1}E_{i,t}^{r}+\theta_{2}S_{j,t}+\theta_{3}D_{t}+u_{i}+u_{j}+\epsilon_{i,t}, \end{array}$$ (22)

The class-level inquiry representation and prospective classroom analysis model are summarized in Equations 20–22 where $E_{i,t+1}^{r}$ is the next-stage engagement level in dimension *r*, *D*_*t*_ denotes task difficulty, *u*_*i*_ and *u*_*j*_ are learner- and class-level random effects, and ϵ_*i,t*_ is the residual term. Although the present study uses public datasets for modular feasibility analysis, this formulation provides the basis for future de-identified classroom deployment in vocational product design courses.

The interpretive sequence is therefore evidence first, teacher judgment second, and instructional action third. Aggregation can help teachers identify patterns that warrant attention, but individual support decisions should return to the underlying evidence chain and the teacher's knowledge of the student and task.

## 4 Results

### 4.1 Overview of public-dataset analyses

The experiments are designed to examine PD-EviMEA from six perspectives: multimodal engagement recognition, behavioral-risk proxy analysis, design-process evidence modeling, teacher-rubric proxy analysis, expert-scoring proxy analysis, and ecological classroom engagement recognition. Because there is no single public dataset that simultaneously contains vocational product design classroom video, learning logs, CAD process records, teacher ratings, and engagement labels, this study adopts a modular public-dataset feasibility strategy. The datasets are not merged at the raw-sample level. Instead, each dataset is used to examine a specific functional module of the proposed framework, as illustrated in Figure 2.

The experimental questions are as follows:

1. Can reliability-gated fusion improve balanced multimodal engagement recognition under class imbalance?
2. Can behavior logs and CAD operation sequences provide useful proxy evidence for behavioral engagement, cognitive engagement, and domain-specific design-task evidence?
3. Can evidence-chain feedback preserve human-rating consistency while adding traceability?
4. Can the ecological module recognize classroom group engagement in authentic classroom videos?
5. Which components of PD-EviMEA contribute most to performance, calibration, robustness, and interpretability?

### 4.2 Datasets

Table 2 summarizes the public datasets used in this study. All datasets are used as secondary public data sources. They serve different functional purposes and are not treated as one unified student cohort.

**Table 2:** Public datasets and their functional roles in the experiments.

Dataset	Scale/labels	Functional role	Interpretive boundary
CMOSE	12,197 clips; four-level engagement labels	Main benchmark for multimodal engagement recognition using I3D, BERT, and acoustic features	Engagement-recognition benchmark; not a product design classroom dataset
OULAD	32,593 student-module-presentation samples	Behavioral engagement and academic-risk proxy analysis	Behavior-log proxy; not a direct psychological engagement label
Fusion 360 Gallery	8,626 CAD construction sequences	Product design process evidence modeling	CAD sequence evidence; not a direct student mental-state label
ASAP 2.0	17,307 essays; 7 prompts; human scores	Public teacher-rubric proxy analysis	Writing-rubric proxy; not product design teacher evaluation
SAS-Bench	4,075 expert-annotated answers; 13,976 step-level samples	Expert-scoring and error-cause proxy analysis	Fine-grained assessment proxy; not classroom utility rating
OUC-CGE	7,705 classroom group video clips; low/mid/high labels	Public ecological classroom feasibility evidence	Authentic classroom proxy; not vocational product design classroom intervention

#### 4.2.1 Rationale for modular dataset selection

The six datasets were selected because each provides an observable evidence type required by the proposed framework, while no available public dataset contains all evidence types in one authentic vocational product design classroom. CMOSE was selected because it provides multimodal engagement labels and supports the main reliability-gated fusion test. OULAD was selected because it contains large-scale learning behavior records, allowing behavior-log evidence to be examined without collecting identifiable classroom traces. Fusion 360 Gallery was selected because it provides genuine CAD construction sequences that are directly relevant to product-design process evidence. ASAP 2.0 and SAS-Bench were selected because they include human scoring or expert-scoring information, which makes it possible to test whether feedback and scoring components can remain aligned with human criteria under public benchmark conditions. OUC-CGE was selected because it provides authentic classroom group-engagement videos and therefore offers ecological classroom feasibility evidence, although it is not a vocational product design dataset.

This modular strategy is scientifically appropriate only for proof-of-concept feasibility analysis. The datasets are not pooled, the models are not trained as one cross-dataset learner model, and the results are not interpreted as evidence that PD-EviMEA has been comprehensively validated as a deployable classroom assessment system. The claims that can be made are component-level claims: whether each type of evidence can support a functional part of the framework under reproducible public-data conditions. The claims that cannot be made are classroom-effectiveness claims, product design teacher acceptance claims, longitudinal learning-outcome claims, or direct evidence that the integrated system improves teaching practice in an authentic studio course.

#### 4.2.2 CMOSE

CMOSE contains 12,197 multimodal learning clips with four engagement labels. The label distribution is highly imbalanced: Engage = 8,470, Disengage = 2,209, Highly Engage = 1,171, and Highly Disengage = 347. The official JSON features include I3D, BERT, and acoustic features. Due to feature-file extraction incompatibility, OpenFace-based gaze, head-pose, and facial-action-unit features are not included in the present reproducible experiment.

#### 4.2.3 OULAD

OULAD contains 32,593 student-module-presentation samples and provides virtual learning environment interactions, assessment-related information, and final outcomes. It is used to examine whether behavior logs can provide proxy evidence for behavioral engagement and academic risk.

#### 4.2.4 Fusion 360 gallery reconstruction

This dataset contains 8,626 CAD construction sequences with an average sequence length of 20.03. It is used to model CAD operation transitions, design-stage prediction, and process complexity, thereby supporting product-design-specific design-task evidence.

#### 4.2.5 ASAP 2.0

ASAP 2.0 contains 17,307 student essays from seven writing prompts with human scores. It is used as a public teacher-rubric proxy dataset. The split is stratified by prompt_name + score, with an 80/20 train–test ratio.

#### 4.2.6 SAS-Bench

SAS-Bench contains expert-scored student answers, step-level scores, and error-cause labels. A held-out question split is used to prevent the same question from appearing in both training and testing sets.

#### 4.2.7 OUC-CGE

OUC-CGE contains 7,705 authentic classroom group video clips labeled as low, medium, and high engagement. After extraction, 7,607 videos can be decoded by OpenCV, while 98 high-engagement clips cannot be decoded and are retained with missing visual features handled by imputation. This dataset is used for public ecological classroom feasibility analysis.

### 4.3 Evaluation metrics

For classification tasks, we report Accuracy, Macro-F1, Balanced Accuracy, quadratic weighted kappa (QWK), and expected calibration error (ECE). Macro-F1 is used as a primary metric when class imbalance is present, because it assigns equal importance to each class. QWK is used for ordinal labels, because engagement levels and human scores have ordered structures. ECE measures the discrepancy between model confidence and empirical accuracy:


$$\begin{array}{l} ECE=\overset{B}{\underset{b=1}{\sum }}\frac{\left|B_{b}\right|}{n}|\text{acc}\left(B_{b}\right)-\text{conf}\left(B_{b}\right)|, \end{array}$$ (23)

Expected calibration error is defined in Equation 23 where $B_{b}$ is the *b*-th confidence bin, *n* is the number of samples, $\text{acc}\left(B_{b}\right)$ is the accuracy in the bin, and $\text{conf}\left(B_{b}\right)$ is the average confidence in the bin.

For scoring tasks, we report QWK, Spearman correlation, mean absolute error (MAE), normalized MAE, and root mean squared error (RMSE). For design-process prediction, we report Top-1 and Top-3 accuracy for next-operation prediction, Macro-F1 for stage classification, and MAE/RMSE for complexity estimation. For explanation-oriented evaluation, we report evidence traceability through rule-checked citation coverage and evidence support when applicable. These rule-checked scores are not interpreted as teacher utility ratings; they only indicate whether the generated feedback is grounded in selected textual evidence.

To strengthen practical interpretation, the analysis also reports effect magnitudes as absolute metric differences and relative percentage changes for the main within-task comparisons. In classification and ordinal prediction, these effect magnitudes are used as practical effect-size indicators because the public benchmark tasks differ in label scales, modalities, and available paired predictions. For calibration, practical significance is interpreted as reduction in ECE. For evidence-chain feedback, practical significance is interpreted in terms of added traceability when predictive scores are unchanged.

### 4.4 Analysis settings

For the newly added public proxy analyses, each experiment is repeated with five random seeds: 42, 2,025, 3,407, 7, and 123. We report the mean result and 95% confidence interval. Paired bootstrap tests are used to compare model differences when paired predictions are available. When bootstrap *p* values are close to zero, they are reported as *p* < 0.001 rather than *p* = 0.000. When paired predictions are not available across all baseline settings, the paper avoids unsupported significance claims and instead reports confidence intervals, absolute effects, relative effects, and interpretive boundaries.

For CMOSE, we use the official split and compare probability fusion, learned stacking, and PD-EviMEA reliability-gated fusion. For ASAP 2.0, the train–test split is stratified by prompt and score. For SAS-Bench, held-out question split is used to reduce question-level leakage. For OUC-CGE, we use the full 7,705 clips and report visual-only, metadata-only, and visual+metadata settings to examine the risk of metadata leakage.

### 4.5 Benchmark comparison

Because the datasets used in this study support different modalities and tasks, the comparison focuses on competitive and reproducible baselines rather than a single unified leaderboard. For multimodal engagement recognition on CMOSE, the baselines include mean probability fusion and learned probability stacking. For ecological classroom feasibility analysis on OUC-CGE, the baselines include visual-stat Logistic Regression and metadata-only Random Forest. For rubric-aligned scoring on ASAP 2.0, TF-IDF Ridge scoring is used as the scoring baseline. For SAS-Bench, the full model is compared with a variant without guideline input.

Table 3 provides a consolidated comparison with representative baselines and competitive methods across the public benchmark tasks. Because the datasets differ in labels, modalities, and learning contexts, the table should be read as a module-level comparison rather than a single unified leaderboard. Best values within each task block are highlighted in bold.

**Table 3:** Representative state-of-the-art-oriented and competitive baseline comparison across benchmark tasks.

Task/dataset	Method	Type	Primary metric	Value	Interpretation
CMOSE	I3D+BERT+acoustic random forest	Submodel baseline	Macro-F1	0.4239	Strong branch baseline
CMOSE	Mean probability fusion	Late fusion	Macro-F1/QWK	0.2091/0.0048	Majority-class tendency
CMOSE	Learned probability stacking	Competitive fusion	Macro-F1/QWK	0.4812/0.4178	Learned late fusion
CMOSE	**PD-EviMEA reliability gate**	Proposed	Macro-F1/QWK	**0.5162/0.4216**	Best balanced recognition
OUC-CGE	Visual-stat logistic regression	Linear visual baseline	Macro-F1/QWK	0.5325/0.3915	Ecological baseline
OUC-CGE	Metadata-only random forest	Leakage-control baseline	Macro-F1/QWK	0.5747/0.4074	Metadata signal check
OUC-CGE	PD-EviMEA-eco visual-stat RF	Proposed visual model	Macro-F1/QWK	0.7967/0.7432	Robust visual evidence
OUC-CGE	**PD-EviMEA-eco visual+metadata RF**	Proposed enhanced model	Macro-F1/QWK	**0.8421/ 0.7988**	Best ecological result
ASAP 2.0	TF-IDF Ridge scoring	Rubric scoring baseline	QWK/Spearman	0.6691/0.8041	Scoring head
ASAP 2.0	PD-EviMEA-text evidence chain	Proposed explanation layer	QWK/Spearman	0.6691/0.8041	Same score, added traceability
SAS-Bench	Full PD-EviMEA-SAS	Proposed diagnostic model	QWK/ECS-F1	0.6408/0.3245	Moderate expert agreement
SAS-Bench	w/o Guideline Input	Ablation baseline	QWK	0.6525	Guideline not yet exploited
Fusion 360	Markov transition	Sequence baseline	Top-1/Top-3	0.6901/0.9102	CAD operation prediction
Fusion 360	n-gram Logistic Regression	Stage baseline	Macro-F1	0.5880	Stage transition modeling

Best results within each task block are highlighted in bold.

Table 4 summarizes the practical effect magnitudes of the main comparisons. These values should be read within each dataset and task block rather than as cross-dataset effect sizes.

**Table 4:** Practical effect magnitudes for main module-level comparisons.

Dataset/module	Comparison	Absolute effect	Relative effect	Practical interpretation
CMOSE fusion	PD-EviMEA vs. learned stacking	Macro-F1 Δ = +0.0350	+7.3%	Meaningful improvement in balanced recognition under class imbalance
CMOSE calibration	Full model vs. w/o correction	ECE Δ = −0.0844	65.6% reduction	Substantial improvement in confidence calibration
OUC-CGE visual evidence	Visual-stat RF vs. visual Logistic Regression	Macro-F1 Δ = +0.2642	+49.6%	Strong module-level classroom-video feasibility evidence
OUC-CGE metadata control	Visual+metadata RF vs. metadata-only RF	Macro-F1 Δ = +0.2674	+46.5%	Visual evidence adds information beyond metadata signal
ASAP 2.0 evidence chain	Evidence-chain layer vs. TF-IDF Ridge	QWK Δ = 0.0000	unchanged	Traceability is added without improving or reducing scoring accuracy
SAS-Bench guideline use	Full model vs. w/o guideline	QWK Δ = −0.0117	−1.8%	Current lightweight model does not yet exploit long guidelines effectively

#### 4.5.1 CMOSE multimodal engagement recognition

Table 5 reports the fusion results on CMOSE. Although mean probability fusion obtains relatively high Accuracy, its Macro-F1 and QWK are extremely low, indicating majority-class prediction under severe class imbalance. PD-EviMEA achieves the best Macro-F1 and QWK, suggesting stronger balanced engagement recognition.

**Table 5:** Fusion model comparison on CMOSE.

Model	Acc.	Macro-F1	Bal. Acc.	QWK	ECE
Mean probability fusion	0.6945	0.2091	–	0.0048	–
Learned probability stacking	0.5897	0.4812	–	0.4178	–
PD-EviMEA reliability gate	0.6839	0.5162	0.5557	0.4216	0.0443

**Figure 4:**
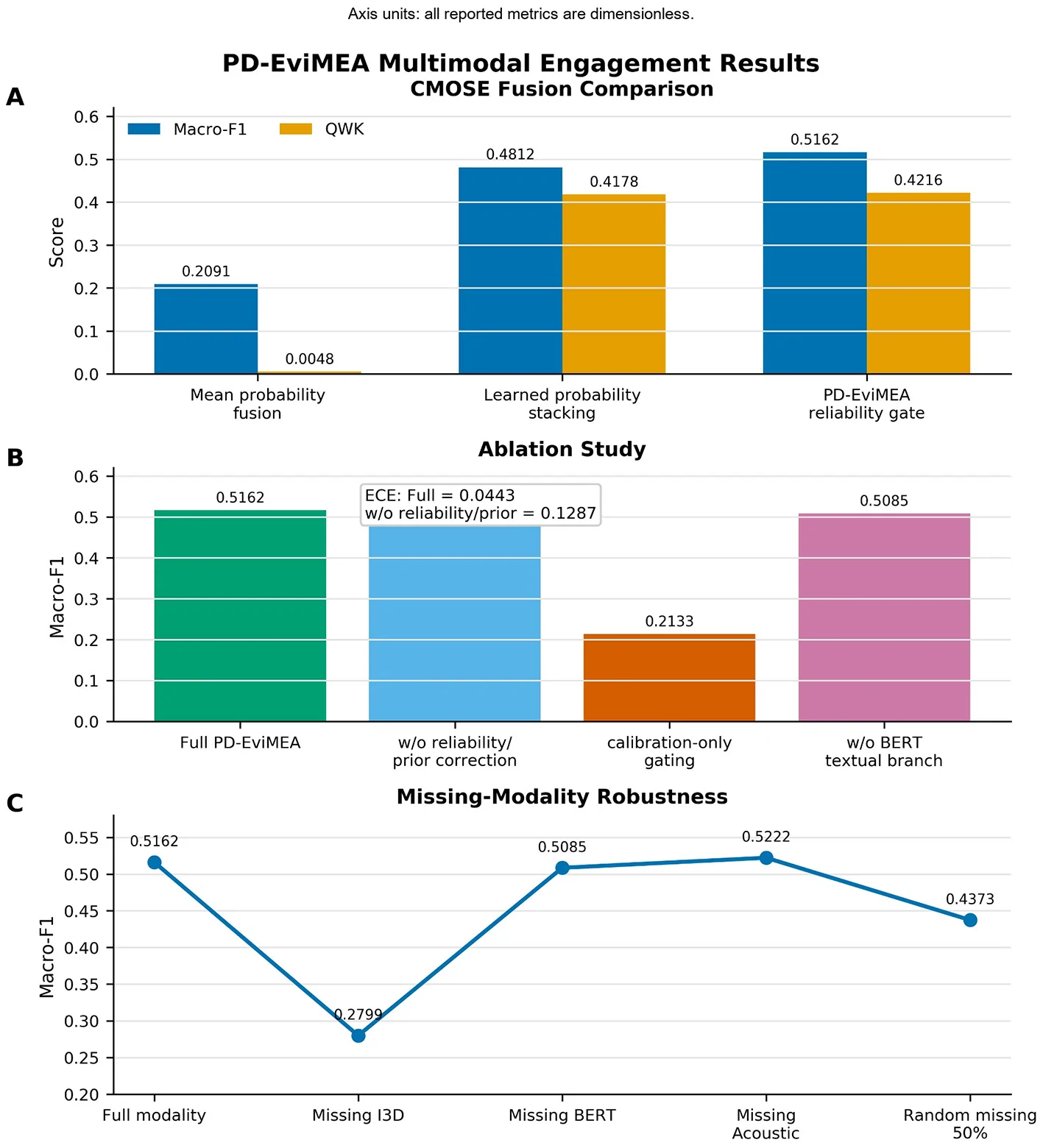
CMOSE multimodal engagement recognition results. **(A)** Compares fusion strategies; **(B)** reports ablation results; **(C)** shows missing-modality robustness. The results indicate that reliability-gated fusion improves balanced engagement recognition, while I3D visual features contribute most strongly to the CMOSE engagement task.

The result, visually summarized in Figure 4A, shows that Accuracy alone is misleading for imbalanced engagement recognition. PD-EviMEA does not simply optimize majority-class prediction; instead, it improves Macro-F1 and QWK, which better reflect its ability to distinguish minority engagement states.

#### 4.5.2 OULAD and Fusion 360 module-level feasibility evidence

Table 6 summarizes the OULAD and Fusion 360 module-level results. OULAD examines the predictive value of behavior-log evidence, while Fusion 360 examines the feasibility of extracting design-process evidence from CAD sequences.

**Table 6:** Behavioral-risk and design-process module-level feasibility evidence.

Dataset/task	Best model and result	Interpretation
OULAD behavior prediction	Logistic Regression: AUC = 0.9855, F1 = 0.9436, Accuracy = 0.9457	Behavior logs provide strong proxy evidence for academic risk and participation, but are not direct psychological engagement labels.
OULAD ordinal proxy	Random Forest: Accuracy = 0.8628, Macro-F1 = 0.8246, QWK = 0.9281	Behavior-related features support ordinal outcome modeling.
Fusion 360 next-operation prediction	Markov transition: Top-1 = 0.6901, Top-3 = 0.9102	CAD operation sequences contain learnable process structures.
Fusion 360 stage recognition	n-gram Logistic Regression: Macro-F1 = 0.5880	CAD sequences can be mapped to design-stage evidence.
Fusion 360 complexity estimation	RandomForest operation-stat regressor: MAE = 0.1659, RMSE = 0.3153	Design-process complexity can be quantified as domain-specific evidence.

#### 4.5.3 Public teacher-rubric proxy analysis

**Figure 5:**
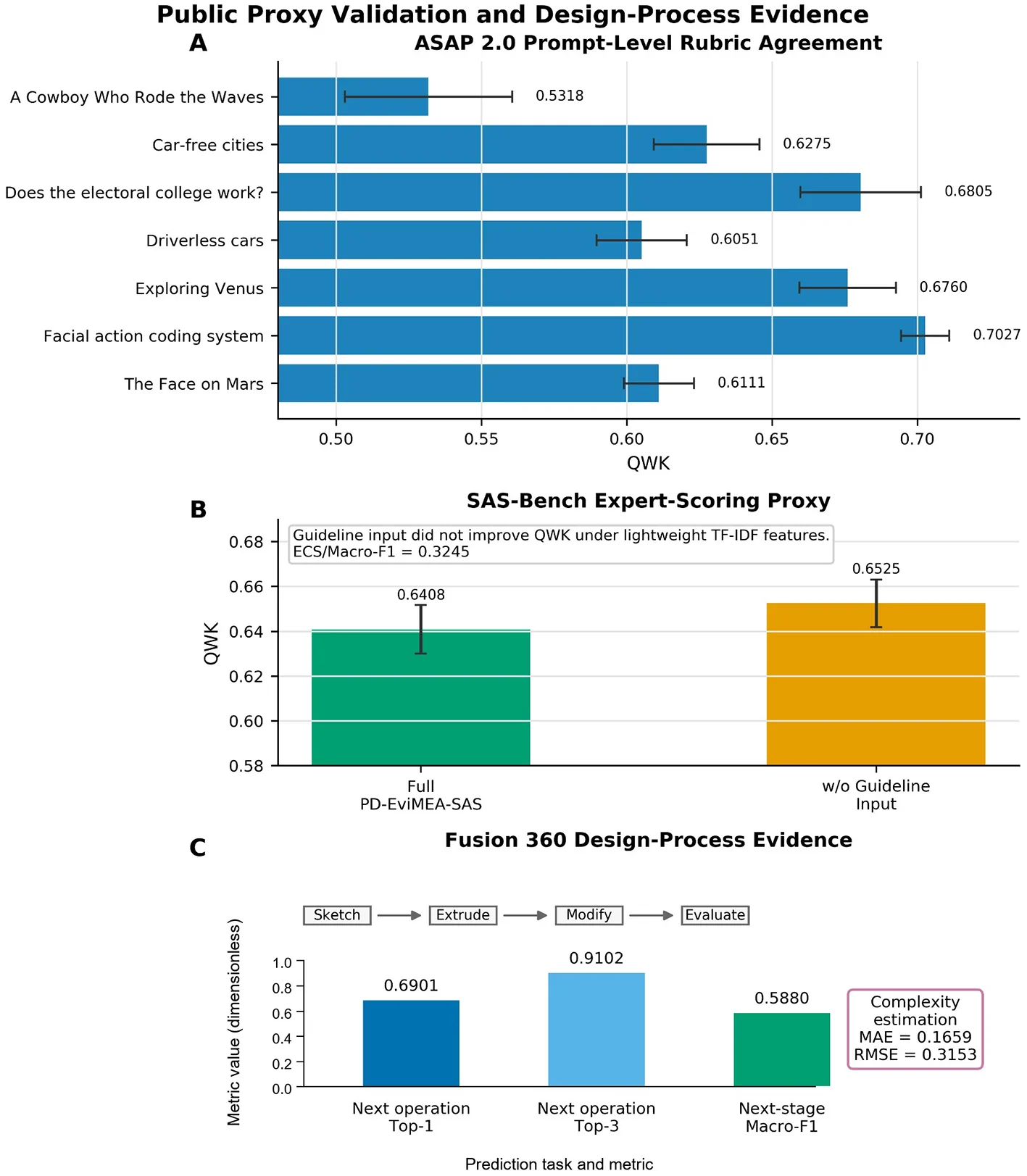
Public proxy analysis and product-design process evidence. **(A)** Reports ASAP 2.0 prompt-level rubric agreement. **(B)** Reports SAS-Bench expert-scoring proxy results. **(C)** Summarizes Fusion 360 CAD process modeling results, supporting the feasibility of using design-process traces as domain-specific evidence for product design learning.

Table 7 reports the ASAP 2.0 teacher-rubric proxy results. The evidence-chain version uses the same scoring head as TF-IDF Ridge scoring; therefore, the predictive scores are identical. The contribution of PD-EviMEA-text in this setting lies in adding sentence-level evidence traceability without degrading scoring performance. Figure 5 summarizes the prompt-level rubric agreement, SAS-Bench expert-scoring proxy results, and Fusion 360 design-process evidence.

**Table 7:** ASAP 2.0 teacher-rubric proxy analysis.

Model	QWK	95% CI	Spearman	MAE
TF-IDF Ridge scoring	0.6691	[0.6639, 0.6743]	0.8041	0.5241
PD-EviMEA-text evidence chain	0.6691	[0.6639, 0.6743]	0.8041	0.5241

Table 8 reports prompt-level QWK values. The results indicate that different writing tasks have different scoring difficulty. Since the split is within-prompt stratified, these results should not be interpreted as cross-prompt generalization.

**Table 8:** Prompt-level QWK results on ASAP 2.0.

Prompt	QWK [95% CI]
A Cowboy Who Rode the Waves	0.5318 [0.5030, 0.5606]
Car-free cities	0.6275 [0.6093, 0.6457]
Does the electoral college work?	0.6805 [0.6598, 0.7012]
Driverless cars	0.6051 [0.5896, 0.6207]
Exploring Venus	0.6760 [0.6594, 0.6926]
Facial action coding system	0.7027 [0.6944, 0.7110]
The Face on Mars	0.6111 [0.5990, 0.6232]

Table 9 reports SAS-Bench expert-scoring proxy analysis. The full model reaches moderate agreement with expert scores. However, the variant without guideline input obtains a slightly higher QWK, suggesting that the current lightweight TF-IDF and structural-feature setting does not fully exploit long scoring guidelines.

**Table 9:** SAS-Bench expert-scoring and diagnostic proxy analysis.

Model	QWK	95% CI	Spearman	Norm-MAE	CCS	ECS/F1
Full PD-EviMEA-SAS	0.6408	[0.6300, 0.6517]	0.6672	0.2074	0.8014	0.3245
w/o guideline input	0.6525	[0.6418, 0.6631]	0.6756	0.2022	–	–

#### 4.5.4 Public ecological classroom feasibility analysis

Table 10 reports the full OUC-CGE results. PD-EviMEA-eco visual-stat RF substantially outperforms the visual-stat Logistic Regression baseline. The visual+metadata RF model obtains the best performance, while metadata-only RF is reported to examine possible metadata leakage.

**Table 10:** Public ecological classroom feasibility analysis on OUC-CGE.

Model	Macro-F1	95% CI	QWK	ECE
Visual-stat logistic regression	0.5325	[0.5275, 0.5375]	0.3915	–
PD-EviMEA-eco visual-stat RF	0.7967	[0.7853, 0.8080]	0.7432	–
Metadata-only RF	0.5747	[0.5687, 0.5806]	0.4074	–
PD-EviMEA-eco visual+metadata RF	0.8421	[0.8362, 0.8481]	0.7988	0.1025

**Figure 6:**
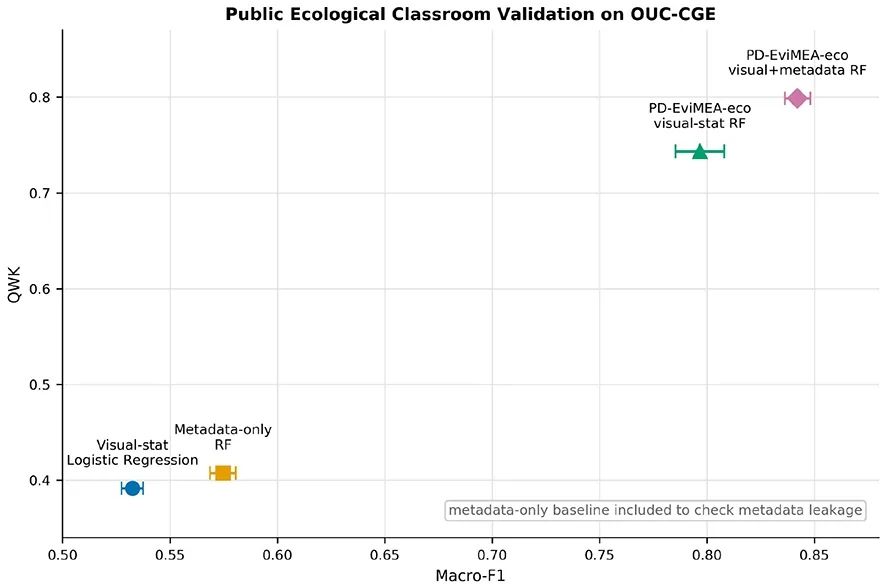
Public ecological classroom feasibility analysis on OUC-CGE. PD-EviMEA-eco visual-stat RF and visual+metadata RF achieved higher Macro-F1 and QWK than the logistic regression and metadata-only baselines. The metadata-only baseline was included to examine potential metadata leakage.

The visual-only result is treated as the main robust evidence, as also shown in Figure 6, because it does not depend on metadata. The metadata-only baseline is higher than random-level prediction but clearly lower than visual-stat RF and visual+metadata RF, suggesting that metadata contains some predictive signal but cannot explain the main model performance by itself. Future work should further perform clean-metadata sensitivity analysis by removing file paths, file names, source identifiers, class-folder identifiers, and decoding-status indicators.

### 4.6 Ablation study

Table 11 reports ablation results on CMOSE. Removing class reliability and prior correction reduces Macro-F1 and substantially worsens ECE, as shown in Figure 4B. The calibration-only gating variant performs poorly, indicating that calibration alone is insufficient for robust multimodal fusion under severe class imbalance. Removing the BERT textual branch has a smaller effect, suggesting that the visual I3D branch contributes most strongly in this dataset.

**Table 11:** Ablation study on CMOSE.

Model variant	Macro-F1	QWK	ECE
Full PD-EviMEA	0.5162	0.4216	0.0443
w/o class reliability/prior correction	0.4998	–	0.1287
Calibration-only gating	0.2133	–	–
w/o BERT textual branch	0.5085	–	–

### 4.7 Missing-modality robustness

Table 12 reports the missing-modality analysis on CMOSE. The results in Figure 4C indicate that I3D visual features are the most important modality in the current CMOSE setting. The model remains relatively stable when BERT or acoustic features are removed, while performance decreases substantially when I3D features are missing.

**Table 12:** Missing-modality analysis on CMOSE.

Condition	Macro-F1
Full modality	0.5162
Missing I3D	0.2799
Missing BERT	0.5085
Missing acoustic	0.5222
Random missing 50%	0.4373

This analysis prevents overclaiming robustness. PD-EviMEA can handle incomplete modalities, but its performance still depends on the educational relevance and quality of the remaining modalities. In CMOSE, visual behavior is the dominant engagement signal.

### 4.8 Generalization analysis

The generalization analysis is conducted at three levels. First, cross-dataset functional generalization is examined through the modular feasibility strategy. CMOSE examines multimodal engagement recognition, OULAD examines behavior-log risk modeling, Fusion 360 examines design-process evidence extraction, ASAP 2.0 and SAS-Bench examine human-score proxy alignment, and OUC-CGE examines ecological classroom engagement recognition. This design does not claim that the same model is trained on one dataset and directly transferred to all others; instead, it evaluates whether each functional component of PD-EviMEA can be supported by public evidence.

Second, task-level generalization is examined in ASAP 2.0 and SAS-Bench. ASAP 2.0 reports prompt-level QWK values under within-prompt stratified split, showing that the scoring module is stable across different prompts but not yet validated for unseen-prompt transfer. SAS-Bench uses held-out question split, which is stricter than random answer-level splitting and reduces question leakage.

Third, ecological generalization is examined on OUC-CGE. Unlike online-only or laboratory-based datasets, OUC-CGE contains authentic classroom group engagement videos. The strong visual-stat RF result suggests that PD-EviMEA can capture engagement-related visual patterns in real classroom contexts. However, because OUC-CGE is not a vocational product design dataset, this result should be interpreted as public ecological classroom feasibility evidence rather than direct validation in a product design studio course.

### 4.9 Explanation analysis

The explanation analysis focuses on whether model outputs can be linked to observable evidence. In the evidence-chain design, each feedback statement is required to cite or correspond to at least one evidence unit. On ASAP 2.0, the evidence-chain version preserves the same scoring performance as TF-IDF Ridge scoring while adding sentence-level traceability. Thus, the improvement lies in auditability rather than scoring accuracy. On Fusion 360, CAD operation sequences provide evidence for engagement-related design activity, including operation continuity, sequence length, modeling complexity, and stage transition. On OULAD, behavior logs support explanations about academic-risk or behavioral-engagement proxies. On CMOSE and OUC-CGE, visual features support engagement-state recognition, but explanations should be interpreted carefully because visual statistics alone cannot reveal the full psychological meaning of engagement.

Four evidence-chain cases are generated in the experiment: one from CMOSE, two from OULAD, and one from Fusion 360. Each case contains a learning context, engagement diagnosis, supporting evidence chain, and teaching inquiry suggestion. These cases illustrate how PD-EviMEA transforms raw data into pedagogically discussable evidence, but they do not replace teacher judgment. They also do not constitute human validation of interpretability. Their purpose is to check whether the system can produce evidence-linked statements under rule-based traceability constraints. Teacher-perceived usefulness, interpretability, trust, usability, educational relevance, and agreement with product design teachers remain future validation criteria.

### 4.10 Error analysis

The main error patterns should be interpreted at the module level rather than as direct diagnoses of individual classroom learners. In CMOSE, the strongest vulnerability is loss or weakness of the visual signal: removing the I3D branch reduces Macro-F1 from 0.5162 to 0.2799, whereas removing BERT or acoustic features has much smaller effects. This pattern suggests that misclassification is most likely when visual behavior is ambiguous, weakly captured, or unavailable. The calibration results also show that reliability and prior correction reduce overconfident errors, with ECE increasing from 0.0443 to 0.1287 when this correction is removed.

For behavior-log and design-process modules, errors mainly arise from the fact that public labels are proxies for engagement-related evidence rather than direct psychological measurements. OULAD logs can indicate participation and academic-risk patterns, but they cannot reveal the motives or emotions behind those patterns. Fusion 360 sequences can capture operation continuity, design-stage transition, and construction complexity, but they cannot by themselves determine whether a long or complex sequence reflects productive engagement, trial-and-error struggle, or task difficulty. In the scoring modules, ASAP 2.0 shows that the evidence-chain layer adds traceability without changing the regression head, while SAS-Bench indicates that lightweight lexical and structural features do not yet use long scoring guidelines effectively. In OUC-CGE, the predictive signal from metadata-only models indicates a possible contextual confounding risk; therefore, visual-stat models are reported as the more conservative ecological result, and future work should use grouped splits and stronger metadata controls.

### 4.11 Parameter and configuration analysis

The main configurable components of PD-EviMEA include the modality confidence weight λ, the number of retained evidence units *K*_*e*_, the use of reliability/prior correction, and the inclusion of metadata in ecological validation. The ablation results show that removing reliability and prior correction worsens ECE from 0.0443 to 0.1287, indicating that this configuration is important for confidence calibration. The missing-modality results further show that modality configuration strongly affects performance: removing I3D reduces Macro-F1 from 0.5162 to 0.2799, whereas removing BERT or acoustic features has much smaller effects.

In OUC-CGE, metadata inclusion improves Macro-F1 from 0.7967 to 0.8421, but metadata-only RF reaches 0.5747, suggesting that metadata carries some predictive signal. Therefore, metadata should be treated as an auxiliary configuration rather than the primary evidence source. The visual-stat RF result is used as the main robust ecological result.

Table 13 summarizes the main parameter and configuration effects observed in the experiments. The current evidence supports the importance of reliability/prior correction, modality selection, and metadata controls; it also indicates that future work should conduct finer-grained sweeps over continuous hyperparameters such as λ and *K*_*e*_.

**Table 13:** Parameter and configuration analysis.

Configuration	Compared setting	Observed effect	Interpretation
Reliability/prior correction	Full vs. w/o correction on CMOSE	ECE 0.0443 → 0.1287	Correction improves confidence calibration
Dominant visual modality	Full vs. missing I3D on CMOSE	Macro-F1 0.5162 → 0.2799	I3D visual evidence is critical in CMOSE
Text modality	Full vs. missing BERT on CMOSE	Macro-F1 0.5162 → 0.5085	Text has smaller effect in current setting
Metadata inclusion	Visual-stat vs. visual+metadata on OUC-CGE	Macro-F1 0.7967 → 0.8421	Metadata helps but requires leakage control
Evidence-chain layer	TF-IDF Ridge vs. evidence-chain ASAP	QWK unchanged at 0.6691	Traceability improves without changing scoring head
Guideline input	Full SAS vs. w/o guideline	QWK 0.6408 vs. 0.6525	Lightweight features cannot exploit long guidelines

Future parameter analysis should systematically vary λ and *K*_*e*_ and evaluate their influence on Macro-F1, ECE, teacher-perceived cognitive load, and evidence faithfulness.

### 4.12 Computational cost and deployment considerations

The present experiments focus on public-dataset feasibility rather than classroom deployment benchmarking, but several practical cost considerations can be identified. The most computationally expensive step is feature extraction for video or audio-visual data, because classroom video features usually require offline preprocessing or dedicated GPU resources. Once modality features are extracted, the reliability-gated fusion, random-forest, logistic-regression, ridge-regression, and evidence-ranking components used in the present analyses are comparatively lightweight and can be executed at the level of learning episodes rather than frame-by-frame real-time inference. Evidence-chain construction is linear in the number of candidate evidence units before top-*K*_*e*_ selection, so its classroom cost can be controlled by limiting the evidence budget shown to teachers.

For practical use, PD-EviMEA should therefore be deployed as an asynchronous teacher-support workflow rather than as a continuous surveillance or automatic grading system. Video and CAD traces can be processed after a lesson or design milestone; teachers can then inspect a small number of high-confidence evidence units before making instructional decisions. Future work should report hardware configuration, feature-extraction time, memory use, and teacher-dashboard response time when an ethically approved classroom version is implemented.

### 4.13 Limitations and future validation needs

Several limitations and next-step validation needs should be noted. First, the experiments are based on public datasets and proxy analyses. This public-data strategy is appropriate for early-stage framework development because it avoids collecting identifiable classroom data before institutional review; however, classroom implementation and expert-teacher review remain important next-stage validation tasks. Second, CMOSE experiments use I3D, BERT, and acoustic features, but do not include OpenFace gaze, head-pose, and facial-action-unit features due to file extraction incompatibility. Third, OULAD and Fusion 360 provide useful behavioral and design-process evidence, but their labels are proxies rather than direct psychological engagement labels. Fourth, ASAP 2.0 and SAS-Bench support teacher-rubric and expert-scoring proxy analysis under public benchmark conditions, while direct evaluation by product design teachers should be examined in a future classroom-facing study. Fifth, OUC-CGE metadata-only results indicate possible metadata-related prediction signals, requiring stronger clean-metadata and grouped-split sensitivity analyses in future work. Sixth, the evidence-chain cases have not yet been evaluated by teachers; therefore, interpretability is supported only by rule-based traceability checks and not by human judgments of usefulness, trust, or usability. Finally, the current SAS-Bench results show that long scoring guidelines are not effectively used by the lightweight TF-IDF and structural-feature model, suggesting that stronger semantic encoders are needed for guideline-aware diagnosis. These limitations define the next phase of the research program: the present paper provides a construct-informed proof-of-concept framework and preliminary public evidence, while future ethically approved studies should examine classroom use, teacher review, and longitudinal learning outcomes.

A staged validation plan is needed to evaluate the complete framework under authentic educational conditions. The first stage should involve co-design sessions and a small prospective pilot with product design teachers to refine the local evidence ontology, reporting frequency, uncertainty displays, and teacher-override workflow. The second stage should evaluate the full pipeline within the same student cohort by synchronizing classroom video, learning-platform records, design reflections, CAD histories, teacher ratings, and task milestones across a complete studio project. This study should use temporally separated training and evaluation periods, learner- or class-grouped splits, preregistered outcomes, and analyses of calibration, missing modalities, subgroup performance, and agreement between evidence-chain claims and teacher judgments. The third stage should use a longitudinal, multi-class or multi-institution design to examine whether teacher-reviewed evidence supports formative decisions, student support, sustained engagement, and design learning over time.

The modular evidence reported here cannot estimate full-system performance, interactions among modalities collected in the same classroom, teacher adoption, student responses to feedback, or the causal effect of the framework on learning. Its generalizability is therefore limited to the feasibility of the tested components under their respective public-dataset conditions. Even a future single-site classroom study would establish local ecological validity rather than universal transportability; broader generalization would require replication across institutions, course levels, studio tasks, learner groups, and data-collection arrangements.

### 4.14 Summary of experimental findings

The experiments provide four forms of preliminary public evidence. First, PD-EviMEA improves balanced multimodal engagement recognition on CMOSE, especially in terms of Macro-F1 and QWK under class imbalance. Second, OULAD and Fusion 360 suggest that behavior logs and CAD operation sequences can provide meaningful proxy evidence for behavioral engagement and engagement-related design activity. Third, ASAP 2.0 and SAS-Bench show that evidence-chain and diagnostic modules can be aligned with public human-scoring benchmarks, although guideline-aware reasoning and error-cause diagnosis remain challenging. Fourth, OUC-CGE results indicate that PD-EviMEA-eco can recognize classroom group engagement in authentic classroom videos, providing public ecological feasibility evidence for the framework. Together, these findings suggest that PD-EviMEA is a traceable and modular multimodal assessment framework that connects engagement recognition, design-process analytics, and evidence-based teaching inquiry.

## 5 Discussion

### 5.1 Multidimensional engagement as construct-informed evidence

The findings should be interpreted primarily in relation to the educational-psychological problem of assessing multidimensional engagement in complex learning environments. Prior engagement research has emphasized that behavioral participation, emotional involvement, cognitive effort, self-regulation, and agency are related but not identical aspects of learning (Fredricks et al., 2004; Pekrun, 2006; Zimmerman, 2002; Reeve and Tseng, 2011; Bond et al., 2020). The present study extends this line of work by showing how heterogeneous traces in studio-based design learning can be organized as evidence for engagement interpretation. In this sense, the contribution is not that a computational model can directly observe students' inner psychological states. Rather, the framework makes explicit which observable traces may support teacher interpretation of engagement and where those interpretations remain uncertain.

This construct-informed view is particularly important for design learning. Studio work often requires sustained ideation, iterative artifact modification, artifact construction, reflection, and response to critique. These activities are not fully captured by generic indicators such as login frequency, click counts, or visible classroom participation. The design-process evidence used in PD-EviMEA therefore functions as a complementary, domain-sensitive evidence layer that can corroborate or qualify interpretations of engagement-related persistence, cognitive elaboration, self-regulation, and agency. It is not constitutive of the multidimensional engagement construct and should not be interpreted as a general fourth engagement dimension. This distinction helps connect multimodal learning analytics with construct validity rather than treating feature extraction as equivalent to psychological measurement.

### 5.2 Implications for studio-based design education

Although vocational product design provides the motivating context for this study, the framework has implications for wider design education. Many studio-based fields, including industrial design, architecture, digital media design, engineering design, maker spaces, medical skills laboratories, laboratory education, and art-and-design education, share a project-based structure in which learning is expressed through artifacts, design decisions, peer critique, procedural practice, and iterative refinement. The evidence-chain approach may therefore be useful beyond vocational product design when the local evidence units are adapted to the learning tasks and assessment culture of a particular studio or practice-based learning environment.

At the same time, generalization should be understood cautiously. A design-process trace has pedagogical meaning only in relation to the task, the stage of the project, the teacher's expectations, and the student's prior learning trajectory. For example, a short computer-aided design sequence may indicate low persistence in one task but efficient expert-like modeling in another. Thus, the proposed framework is best understood as a structure for organizing interpretable evidence, not as a universal scoring rule for all design classrooms. Future generalization studies should therefore begin by reconstructing the local evidence ontology of each domain. In engineering design, the evidence units may emphasize prototype testing, constraint trade-offs, and iteration logs. In architecture studios, they may emphasize spatial reasoning, critique response, and representational refinement. In laboratory education, they may emphasize procedural accuracy, safety-related behavior, and interpretation of experimental observations. Without this local construct mapping, transferring the same evidence chain to another field would risk technical portability without educational validity.

### 5.3 Teacher agency and evidence-based teaching inquiry

A second implication concerns the role of teachers in technology-supported assessment. Prior work on learning analytics and explainable educational artificial intelligence has argued that data systems should support teacher interpretation rather than replace pedagogical judgment (Schildkamp, 2019; Khosravi et al., 2022; Kasneci et al., 2023; Miao and Holmes, 2023). The evidence-chain design contributes to this agenda by requiring diagnostic statements to be linked to observable evidence units. This makes the output inspectable and contestable: teachers can accept, revise, or reject the suggested interpretation based on their knowledge of the learner, the task, and the classroom context.

This design also reframes prediction as part of an inquiry cycle. Instead of asking whether a model can automatically label a student as engaged or disengaged, the framework asks what evidence is available, how reliable that evidence is, what interpretation it supports, and what teaching question should be examined next. This orientation is aligned with evidence-based teaching inquiry because model output becomes a prompt for professional reflection rather than a final assessment decision.

The educational psychology contribution of this design lies in how it can support formative assessment and teacher cognition. Formative assessment requires evidence about where learners are, what learning goals they are moving toward, and what instructional response may help close the gap (Black and Wiliam, 1998; Hattie and Timperley, 2007). Evidence chains can support this process by making the basis of an engagement diagnosis explicit: a teacher can inspect whether the claim is grounded in participation behavior, design revision, reflective explanation, or classroom interaction. This can change pedagogical decision-making from a single label-oriented judgment to a more diagnostic inquiry: whether to invite a quiet but cognitively engaged student into critique, whether to support a learner who is active but not revising designs, or whether to adjust a studio task that produces broad disengagement evidence across a group.

More specifically, teachers could use the evidence at three decision points. Before a milestone, a class-level pattern could identify groups that may need an additional demonstration, a clearer design constraint, or a structured peer-critique prompt. During formative review, a teacher could open an individual evidence chain to compare participation, reflection, and design-process records before deciding whether to ask a diagnostic question, provide affective reassurance, model a CAD procedure, or prompt the student to explain a design rationale. After an intervention, the teacher could examine subsequent evidence windows to determine whether the support was followed by renewed participation, deeper explanation, or more purposeful artifact development. These uses convert the output into a hypothesis for instructional action and follow-up, rather than a label attached to the student.

The same logic can support differentiated student assistance. Low visible participation accompanied by detailed reflection may call for an invitation to contribute rather than remediation; frequent platform activity with little conceptual development may call for strategy-focused feedback; frustration cues combined with repeated unsuccessful operations may justify timely scaffolding; and a sudden class-wide decline may indicate a task-design problem rather than individual disengagement. Because such interpretations remain context dependent, the dashboard should show the source, time, confidence, and alternative explanation for each claim and allow teachers to annotate, reject, or postpone action.

The framework may also reduce the cognitive burden of interpreting heterogeneous traces. Teachers often hold rich contextual knowledge, but classroom video, learning logs, textual reflections, and CAD operation histories are difficult to inspect together. By organizing traces into evidence units, the model can help teachers notice patterns, question uncertain interpretations, and adapt instruction while retaining professional authority. In this sense, the framework aligns with educational psychology theories of engagement, self-regulation, and feedback because it treats assessment as a situated interpretive process rather than as automated psychological classification.

### 5.4 Methodological boundaries and future validation

The present evidence is best understood as early-stage public-dataset-based feasibility evidence. The study uses public datasets to examine different functional components of the framework, but these datasets were not collected from the same vocational product design classroom. Therefore, the results provide modular and proxy evidence for feasibility rather than a direct classroom intervention test. The behavioral-log analyses support the use of activity traces as proxy evidence, the design-process analyses support the feasibility of extracting domain-specific process indicators, and the teacher-rubric and expert-scoring analyses support the traceability of feedback under public benchmark conditions. These results establish a foundation for classroom-facing validation, where the central questions will concern teacher usefulness, student engagement development, and instructional decision-making in authentic studio contexts. They do not establish that the full pipeline will retain the same accuracy, calibration, interpretability, or practical value when all modalities interact within one classroom.

This boundary is not merely a limitation of sample access; it is also a construct-validity issue. Engagement is context-sensitive, and multimodal traces require interpretation against the learning task, classroom norms, and teacher expectations. Accordingly, the next stage of this research should collect de-identified classroom data under ethical approval, involve product design teachers in judging evidence-chain usefulness, and evaluate whether teacher-reviewed feedback supports students' sustained engagement, design iteration quality, and self-regulated learning over time.

Human validation is especially important for the evidence-chain component. A future teacher-review study should ask product design teachers to rate the usefulness, interpretability, trustworthiness, usability, educational relevance, and actionability of evidence-chain feedback. It should also examine agreement between teacher interpretations and AI-generated evidence claims, the reasons for teacher disagreement, and the extent to which evidence chains help teachers plan formative responses. This teacher-review phase should be followed by a prospective classroom study in which the complete pipeline is evaluated on synchronized data from the same course and the same learners. Without such evaluation, the present study can only claim traceability and modular feasibility, not demonstrated classroom utility or educational effectiveness.

### 5.5 Ethical and responsible use considerations

AI-supported engagement assessment raises ethical issues that go beyond model performance. Multimodal classroom data may include video, audio, posture, facial expression, platform logs, text, and design artifacts, all of which can reveal sensitive information about students' learning behavior and classroom participation. Future classroom studies should therefore use institutional ethical approval, informed consent, data minimization, de-identification, secure storage, and clearly specified retention periods. Students and teachers should know what data are collected, how evidence units are generated, who can inspect the output, and how they can contest or correct an interpretation.

Bias and fairness also require explicit attention. Engagement models can be affected by uneven visibility, camera placement, disability-related behavior differences, language proficiency, cultural display rules, prior design experience, or unequal access to digital tools. For this reason, PD-EviMEA should not be used to rank students or make high-stakes decisions without teacher review. Future evaluations should examine subgroup performance, missing-modality patterns, calibration across learner groups, and whether teacher-facing evidence reduces or amplifies existing biases (Jin et al., 2024; Miao and Cukurova, 2024; Yan et al., 2024b).

Finally, the framework should be positioned as responsible decision support rather than continuous monitoring. The pedagogical goal is to help teachers inspect evidence and plan formative support, not to intensify surveillance. A classroom implementation should limit the granularity and frequency of reporting, foreground uncertainty, allow an “insufficient evidence” output, and make teacher override a normal part of the workflow.

### 5.6 Contributions relative to existing work

Relative to existing student engagement research, this study contributes a way to operationalize multidimensional engagement in a studio context where evidence is distributed across behavior, text, visual participation, and artifact-making processes. Relative to multimodal learning analytics, it foregrounds construct interpretation, modality reliability, and teacher review rather than only predictive accuracy. Relative to explainable educational artificial intelligence, it moves from *post-hoc* feature importance toward evidence units that can be inspected as pedagogical claims. Relative to design-process analytics, it connects computer-aided design sequences and revision behavior to educationally meaningful engagement interpretation rather than treating them only as design reconstruction or automation data.

Taken together, the findings suggest that a traceable multimodal framework can help bridge educational psychology and learning analytics when its outputs are presented as evidence for teacher-led inquiry rather than as autonomous psychological diagnoses. The strongest current contribution is therefore conceptual and methodological: PD-EviMEA offers a construct-aware structure for organizing engagement evidence in studio-based design learning, while the empirical results provide preliminary public-dataset-based support for the feasibility of its components.

## 6 Conclusion

This study proposed PD-EviMEA as a proof-of-concept evidence-chain-constrained multimodal assessment framework for student engagement in studio-based design learning, with vocational product design used as the motivating context. The framework was designed to address a central limitation in existing engagement assessment: students' engagement in studio learning is distributed across heterogeneous traces, including classroom video, learning behavior logs, textual explanations, reflective writing, and computer-aided design process sequences. By linking behavioral, affective, and cognitive engagement with contextualized design-process evidence, PD-EviMEA connects educational psychology constructs with domain-specific evidence in design education. Methodologically, the framework integrates modality-specific encoding, reliability-gated fusion, evidence-unit construction, and evidence-chain-constrained feedback generation, thereby transforming multimodal learning traces into traceable and pedagogically interpretable evidence for human-in-the-loop teaching inquiry.

The experimental results provide preliminary public-dataset-based evidence for the feasibility of selected framework components. On CMOSE, PD-EviMEA improved balanced multimodal engagement recognition, achieving Macro-F1 = 0.5162, QWK = 0.4216, and ECE = 0.0443. The OULAD results showed that learning behavior logs can provide useful proxy evidence for behavioral engagement and academic risk, while the Fusion 360 experiments indicated that computer-aided design operation sequences, design-stage transitions, and process complexity can be modeled as domain-specific evidence for engagement-related design activity. In public teacher-rubric and expert-scoring proxy analyses, ASAP 2.0 results showed that the evidence-chain version preserved rubric-aligned scoring performance while adding sentence-level traceability, and SAS-Bench results indicated moderate agreement with expert scoring, although long guideline utilization and fine-grained error-cause diagnosis remain challenging. The OUC-CGE analysis on 7,705 authentic classroom group video clips further provided ecological classroom evidence for group engagement recognition, with the visual-stat RF model reaching Macro-F1 = 0.7967 and QWK = 0.7432, and the visual+metadata RF model reaching Macro-F1 = 0.8421 and QWK = 0.7988. Together, these findings suggest that PD-EviMEA can connect multimodal engagement recognition with evidence-based teaching inquiry when its outputs are treated as teacher-reviewable evidence rather than autonomous psychological diagnoses.

The evidence base also identifies a clear future validation agenda. Because the public datasets were not collected from the same vocational product design classroom, the findings should be interpreted as modular and proxy evidence for the framework rather than as a completed classroom intervention study or validation of a deployable classroom assessment system. The current findings cannot establish full-system performance, educational effectiveness, or transfer across institutions and studio tasks. Future work should therefore examine the framework with synchronized, de-identified studio-course data from the same learner cohort, product design teacher review, and longitudinal classroom outcomes. In addition, the OUC-CGE metadata-only baseline suggests the need for stronger clean-metadata sensitivity analyses and grouped classroom-level validation, while the SAS-Bench results point to the value of stronger semantic encoders for guideline-aware diagnosis.

Future research will proceed in four directions. First, following teacher co-design and a small pilot, synchronized data from authentic vocational product design courses should be collected under appropriate ethical approval to evaluate the full PD-EviMEA pipeline within the same cohort and across sketching, CAD modeling, prototype presentation, peer critique, and teacher-feedback episodes. Second, stronger multimodal and semantic encoders should be incorporated to improve guideline-aware scoring, design-reflection understanding, and fine-grained diagnostic feedback. Third, the evidence-chain mechanism should be embedded into a teacher-facing dashboard so that teachers can inspect evidence units, adjust model-generated diagnoses, record the rationale for instructional decisions, and review subsequent evidence after an intervention. Fourth, longitudinal and multi-site classroom studies are needed to examine whether evidence-chain-based support can improve students' sustained engagement, design development, and teachers' evidence-based inquiry over time. Such studies should include teacher ratings of usefulness, interpretability, trust, usability, educational relevance, and agreement with evidence-chain claims, together with calibration, subgroup, missing-modality, and cross-context analyses.

From the perspective of the broader evidence-based teaching inquiry agenda, the present study should be understood as a student-engagement assessment component rather than a complete implementation of a full teaching and research system. It provides a construct-informed and methodological basis for future work that connects multimodal evidence generation with iterative teaching improvement in studio-based design education.

In summary, this study provides a methodological foundation for connecting multidimensional engagement assessment, design-process analytics, and evidence-based teaching inquiry in studio-based design education. By emphasizing reliability, traceability, construct caution, and teacher agency, PD-EviMEA offers a feasible route for moving from black-box engagement prediction toward interpretable and evidence-grounded assessment support.

## Data Availability

The original contributions presented in the study are included in the article/supplementary material, further inquiries can be directed to the corresponding author.
